# Prognosis prediction with the IHC3 score in patients with node-negative, hormone receptor-positive, HER2-negative early breast cancer

**DOI:** 10.1016/j.esmoop.2024.103963

**Published:** 2024-10-26

**Authors:** K. Seitz, C. Goossens, H. Huebner, P. Gass, S. Uhrig, F. Heindl, J. Emons, M. Ruebner, D. Anetsberger, A. Hartmann, M.W. Beckmann, R. Erber, C.C. Hack, P.A. Fasching, L. Häberle

**Affiliations:** 1 Department of Gynecology and Obstetrics, Universitätsklinikum Erlangen, Friedrich-Alexander-Universität Erlangen-Nürnberg (FAU), Erlangen; 2 Comprehensive Cancer Center Erlangen-EMN (CCC-ER-EMN), Erlangen; 3 Institute of Pathology, Universitätsklinikum Erlangen, Friedrich-Alexander-Universität Erlangen-Nürnberg (FAU), Erlangen; 4 Biostatistics Unit, Department of Gynecology and Obstetrics, Universitätsklinikum Erlangen, Friedrich-Alexander-Universität Erlangen-Nürnberg (FAU), Erlangen, Germany

**Keywords:** early breast cancer, IHC3, molecular marker, prognosis, hormone receptor-positive

## Abstract

**Background:**

Prognostication has been used to identify patient populations that could potentially benefit from treatment de-escalation. In patients with hormone receptor-positive (HRpos), human epidermal growth factor receptor 2-negative (HER2neg) early breast cancer (eBC), treatment de-escalation classically involved omitting chemotherapy. With recently developed specialized therapies that require hands-on side-effect management, the therapeutic landscape is changing and therapy decisions are no longer based only on prognosis, but also consider potential side-effects. Therefore, identification of patient groups based on prognostication has gained importance.

**Materials and methods:**

In this retrospective analysis, a population of 2359 node-negative HRpos/HER2neg eBC patients was selected from all patients treated at the University Breast Center of Franconia, Germany between 2002 and 2021. The prognostic value of the IHC3 score (incorporating immunohistochemical measurements of the estrogen and progesterone receptor status and Ki-67) with clinical parameters (lymph node status, tumor stage, grading) regarding invasive disease-free survival (iDFS) and overall survival (OS) was assessed.

**Results:**

IHC3 positively correlated with Ki-67 expression and inversely correlated with hormone receptor expression. IHC3 categorized into quartiles identified patients with a more unfavorable prognosis: 5-year and 10-year iDFS rates for patients in the highest versus the lowest quartile were 84% versus 95% and 70% versus 88%, respectively. A sensitivity analysis of distant disease-free survival showed similar results to those of iDFS. Five-year and 10-year OS rates for patients in the highest versus the lowest quartile were, respectively, 92% versus 97% and 81% versus 92%.

**Conclusions:**

IHC3 is able to define prognostic groups in patients with node-negative, HRpos/HER2neg eBC. Node-negative patients with a high IHC3 score had the worst prognosis, which was comparable to that of node-positive patients described in recent trials. This simple and cost-effective tool could thus potentially aid in identifying patient groups for innovative therapeutic approaches.

## Highlights

• IHC3 identified patient populations with an unfavorable prognosis.
• IHC3 identified node-negative patients with a similar prognosis to node-positive patients.
• IHC3 is a simple and cost-effective tool that can be used for prognostication.

## Introduction

In patients with hormone receptor-positive (HRpos), human epidermal growth factor receptor 2-negative (HER2neg) early-stage breast cancer, prognosis prediction has been applied to determine which patients should be treated with chemotherapy. Patients with a poor prognosis typically receive chemotherapy, while those with a more favorable prognosis may benefit from treatment de-escalation, thereby avoiding chemotherapy-associated side-effects.^1^, ^2^, ^3^ Although the treatment need for patients with a high recurrence risk is not disputed, even in this group there may be patients who would not markedly benefit from chemotherapy or who could remain recurrence-free without chemotherapy.

Identification of patients with a lower recurrence risk and good prognosis, who may benefit from treatment de-escalation, has been done with models employing multigene signatures, known prognostic factors or a combination of both.^4^, ^5^, ^6^, ^7^ Several large clinical trials investigated whether multigene tests could be used to identify patient groups for whom chemotherapy could be safely omitted based on prognosis prediction.^8^, ^9^, ^10^, ^11^, ^12^ The MINDACT and TailorX studies could indeed identify patients with a good prognosis and showed that in postmenopausal patients, chemotherapy followed by adjuvant endocrine therapy is as effective as endocrine therapy alone.^8^, ^9^, ^10^, ^11^, ^12^ Due to lack of new treatments for patients with early-stage HRpos/HER2neg breast cancer during the past two decades, these de-escalation strategies had been the primary advancements. However, with the recent approval of cyclin-dependent kinase 4/6 (CDK4/6) inhibitor abemaciclib for the treatment of high-risk early-stage breast cancer, the therapeutic landscape is changing.^13^^,^^14^ Additionally, selective estrogen receptor degraders (SERDs) are currently also being tested in adjuvant clinical trials for patients with an intermediate to high recurrence risk.^15^ The introduction of these novel substances has created a new scenario, where therapy decisions are not only based on prognosis, but also on the potential additional side-effects of these advanced treatments, thus emphasizing the need for appropriate patient selection. The monarchE study included patients with a high recurrence risk based on tumor size, nodal status, grading and Ki-67.^13^ Nevertheless, it needs to be noted that patients with lower recurrence risks appear to have similar treatment benefits and that a treatment benefit of abemaciclib regardless of Ki-67 status has been described.^13^ Compared to monarchE, the NATALEE study (adjuvant ribociclib) and the adjuvant lidERA study that investigates the oral SERD giredestrant also included patients with a lower recurrence risk.^15^, ^16^, ^17^ Therefore, tools that can easily and accurately determine prognosis might gain importance for patient selection in the near future, especially for node-negative patients.

In addition to multigene tests, the IHC4 score (or IHC3 for the HER2neg population) can also be used as a prognostic model to identify patient groups based on recurrence risk.^18^ This score determines prognosis based on immunohistological staining of the estrogen receptor (ER), the progesterone receptor (PR), Ki-67 and HER2, combined with clinical prognostic factors. IHC3/IHC4 has been shown to perform comparably to multigene tests, especially in node-negative patients.^19^^,^^20^ Since global implementation of the multigene test might be challenging due to their associated costs and feasibility, the IHC3-score, which can be easily and cost-effectively calculated with routinely acquired parameters, might be a promising alternative. Therefore, this study aimed to determine the prognostic value of the IHC3 score in a larger population of patients with node-negative, HRpos/HER2neg early-stage breast cancer.

## Materials and methods

### Patient selection

Patients included in this retrospective study were selected from the entire population of patients with invasive breast cancer treated at the University Breast Center of Franconia, Germany. Documentation of standard immunohistochemistry procedures started in 2002, making patients treated between 2002 and 2021 eligible. Female patients with unilateral, node-negative, HRpos/HER2neg breast cancer without the presence of *de novo* metastases that had all information available to calculate the IHC3 score and had information available on chemotherapy treatment could be included. This resulted in a final patient population of 2359 node-negative patients with HRpos/HER2neg tumors. Supplementary Figure S1, available at https://doi.org/10.1016/j.esmoop.2024.103963, shows a flow diagram for patient selection. Approval for the retrospective analysis was obtained from the Ethics Committee of the Medical Faculty of Friedrich-Alexander-Universität Erlangen-Nürnberg, Erlangen, Germany (application number 297_17 Bc).

### Data collection

Data were obtained as part of a prospective and continuous quality-assurance process. Certified breast cancer centers in Germany are required to document all primary diagnoses, patient and tumor characteristics, tumor board decisions and treatment characteristics, along with follow-up data for invasive disease-free survival (iDFS), distant disease-free survival (DDFS) and overall survival (OS).^21^^,^^22^ These data are prospectively collected and annually audited by the German Cancer Society (Deutsche Krebsgesellschaft) and German Society for Breast Diseases (Deutsche Gesellschaft für Senologie). Histopathological data including tumor size, axillary lymph node status, tumor grade, and ER, PR and HER2 status have to be documented as part of this process. The data are documented from the original pathology reports. All of the data used in this retrospective analysis were obtained from this database.

### Histopathological data

Grading, tumor type, ER status, PR status, HER2 status and proliferation status as assessed by Ki-67 staining have been routinely recorded at the Breast Center since 1995. The pretreatment core biopsies or surgery specimens were stained in clinical routine as follows: ER-α (clone 1D5, 1 : 200 dilution or clone EP1, dilution 1 : 40; DAKO, Denmark); PR (clone pgR636, 1 : 200 dilution; DAKO, Denmark) and Ki-67 (clone MIB-1, 1 : 200 or 1 : 100 dilution; DAKO, Denmark). The percentage of positively stained cells was stated in the pathological reports. The tumors were considered positive for ER and PR if the percentage of positively stained cells was at least 10%. To assess HER2 status, a polyclonal antibody against HER2 (1 : 200 or a 1 : 1000 dilution, DAKO, Denmark) was used and HER2 status was stated as 0, 1+, 2+ or 3+ in accordance with Sauter et al.^23^ Tumors with a score of 0 or 1+ were considered HER2-negative, whereas 3+ tumors were considered positive. Tumors with 2+ staining were tested for gene copy numbers using chromogene *in situ* hybridization. Using a kit with two probes of different colors [ZytoDot, 2C SPEC HER2(ERBB2)/CEN17; Zyto Vision Ltd., Bremerhaven, Germany], the gene copy numbers of HER2 and centromeres of the corresponding chromosome 17 were retrieved. A HER2/CEN17 ratio of ≥2.2 up to 2013 and ≥2 thereafter was considered to represent amplification of HER2. With regard to the Ki-67 assessment, the overall tumor area was reviewed including the invasion front. Additionally, the region with the highest percentage of Ki-67-positive nuclei was evaluated as part of the routine diagnostics. Even weak staining was considered positive.

### Survival outcomes

iDFS was defined as the time from the date of primary breast cancer diagnosis to the earliest date of disease progression (invasive local, regional and distant recurrences; contralateral breast cancer; second non-breast primary cancer; and death from any cause) or the date of censoring. Patients who were lost to follow-up before the maximum observation time frame of 10 years or were invasive disease-free after the maximum observation time were censored at the last date on which they were known to be invasive disease-free or at the maximum observation time. DDFS (events: distant recurrences, death from any cause) and OS were defined in a similar fashion.

### Statistical analysis

The overall IHC3 score, which incorporated immunohistochemical (IHC) measurements (ER, PR, Ki-67) and clinical information (lymph node status: only node-negative in the present study; tumor stage; grading) was calculated for each patient according to Cuzick et al.^18^ Patients were subsequently grouped by quartiles of the IHC3 score values (IHC3-Q1: <81, IHC3-Q2: 81-122, IHC3-Q3: 122-172, IHC3-Q4: ≥172). As the IHC3 score was a continuous variable, the lower-limit digit is included in the quartile, while the upper limit represents a rounded number and the actual digit is not included in the quartile.

The iDFS and OS rates were estimated for these patient groups using the Kaplan–Meier product-limit method. Analyses are presented for the total group of patients, as well as for subgroups treated or not treated with chemotherapy. Furthermore, the prognostic effect was explored in subgroups defined by deciles (IHC3-D1: <40; IHC3-D2: 40-72; IHC3-D3: 72-90; IHC3-D4: 90-105; IHC3-D5: 105-122; IHC3-D6: 122-142; IHC3-D7: 142-160; IHC3-D8: 160-186; IHC3-D9: 186-224; IHC3-D10: ≥224). Decile limits are presented similarly to quartile limits.

Since iDFS includes local recurrences as events that can still be potentially cured, a sensitivity analysis with DDFS as the outcome, which is known to profoundly affect prognosis, was carried out for the IHC3 quartiles.

Calculations were carried out using R (version 3.6.1; R Development Core Team, Vienna, Austria, 2019).

## Results

### Patients and IHC3 distribution

Patient characteristics of the total patient population as well as the subgroups according to IHC3 quartiles are presented in Table 1. Patients were on average 59.3 (±11.7) years old, with most of these node-negative patients having a tumor size classified as pT1 (69.6%) or pT2 (26.5%). Chemotherapy as per physicians’ choice was given to 27.9% of patients. The IHC3 score showed a normal distribution (Supplementary Figure S2, available at https://doi.org/10.1016/j.esmoop.2024.103963).

**Table 1:** Characteristics of the study population by IHC3 score quartiles (IHC3-Q1 to IHC3-Q4)

Characteristic	Total population (*N* = 2359)	IHC3-score			
		IHC3-Q1 (<81) (*n* = 594)	IHC3-Q2 (81-122) (*n* = 585)	IHC3-Q3 (122-172) (*n* = 592)	IHC3-Q4 (≥172) (*n* = 588)
Age at diagnosis, years	59.3 (11.7)	58.3 (10.2)	59.5 (11.5)	60.4 (11.7)	59.1 (13.3)
ER expression (0%-100%)	80.5 (21.8)	86.5 (12.6)	85.0 (13.7)	81.7 (19.0)	68.5 (31.6)
PR expression (0%-100%)	53.2 (34.7)	73.1 (24.6)	65.7 (29.3)	48.6 (33.1)	25.0 (29.9)
Ki-67 expression (0%-100%)	17.5 (16.1)	7.6 (5.2)	12.4 (8.1)	17.8 (12.3)	32.1 (21.3)
Tumor stage					
T1	1641 (69.6)	560 (94.3)	491 (83.9)	373 (63.0)	217 (36.9)
T2	624 (26.5)	34 (5.7)	94 (16.1)	215 (36.3)	281 (47.8)
T3	64 (2.7)	0 (0.0)	0 (0.0)	2 (0.3)	62 (10.5)
T4	30 (1.3)	0 (0.0)	0 (0.0)	2 (0.3)	28 (4.8)
Grading					
G1	613 (26.0)	461 (77.6)	116 (19.8)	30 (5.1)	6 (1.0)
G2	1374 (58.2)	132 (22.2)	461 (78.8)	499 (84.3)	282 (48.0)
G3	372 (15.8)	1 (0.2)	8 (1.4)	63 (10.6)	300 (51.0)
Chemotherapy					
No	1700 (72.1)	545 (91.8)	491 (83.9)	428 (72.3)	236 (40.1)
Yes	659 (27.9)	49 (8.2)	94 (16.1)	164 (27.7)	352 (59.9)

Data are presented as either mean (standard deviation) or *n* (%).

ER, estrogen receptor; G, grade; IHC, immunohistochemical; PR, progesterone receptor.

### Association between IHC3 score and tumor and patient characteristics

Age was consistent between IHC3 subgroups. ER expression was highest in IHC3-Q1 (86.5%) and showed a gradual decrease across IHC3 subgroups (IHC3-Q2: 85.0%, IHC3-Q3: 81.7%, IHC3-Q3: 68.5%). PR expression decreased more severely across subgroups, from 73.1% in IHC3-Q1 to 25.0% in IHC3-Q4. Conversely, Ki-67 expression showed a gradual increase from 7.6% in IHC3-Q1 to 32.1% in IHC3-Q4. The IHC3-Q1 subgroup consisted almost exclusively of T1 tumors (94.3%), whereas higher tumor stages were more frequently present in the higher IHC3 quartiles. A similar distribution was observed for tumor grading, with the higher IHC3 quartile subgroups generally containing tumors with a grading of 2 or 3. While 8.2% of patients in IHC3-Q1 received chemotherapy, this percentage was 59.9% in IHC3-Q4 (Table 1). Comparable results were obtained when subdividing the IHC3 score into deciles (Supplementary Table S1, available at https://doi.org/10.1016/j.esmoop.2024.103963).

### Association between IHC3 score and iDFS

**Figure 1:**
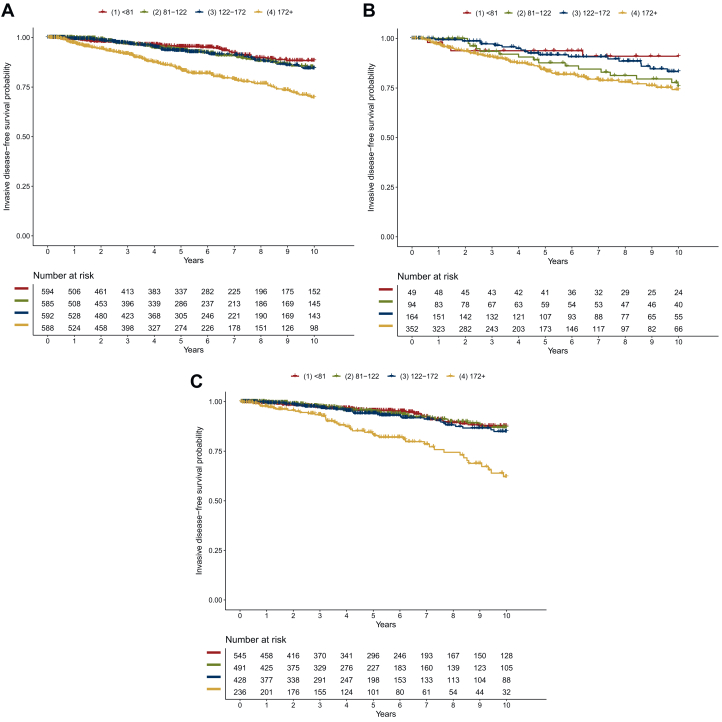
**IHC3 and invasive disease-free survival.** Invasive disease-free survival curves by IHC3 score quartiles (A) in the total patient population, (B) in patients treated with chemotherapy and (C) in patients who did not receive chemotherapy. Red line: IHC3-Q1, green line: IHC3-Q2, blue line: IHC3-Q3: yellow line: IHC3-Q4.

iDFS rates (2-, 5- and 10-year) were consistently lowest in IHC3-Q4 (Table 2). Patients with an IHC3 >172 had a 5-year iDFS rate of 0.84 [95% confidence interval (CI) 0.80-0.87], whereas patients with in IHC3-Q1, IHC3-Q2 or IHC3-Q3 had 5-year iDFS rates of 0.95 (95% CI 0.93-0.97), 0.94 (95% CI 0.91-0.96) and 0.93 (95% CI 0.91-0.96), respectively. Further iDFS rates are provided in Table 2. Kaplan–Meier curves are shown in Figure 1. iDFS rates differed between patients treated or not treated with chemotherapy. While the iDFS rates in patients who did not receive chemotherapy were comparable to the general population (lowest iDFS rates in IHC3-Q4; Table 2 and Figure 1C), patients treated with chemotherapy had the least favorable prognosis in the IHC3-Q4 and IHC3-Q2 subgroups, and IHC3-Q1 and -Q3 had the most favorable iDFS (Table 2 and Figure 1B). Dividing the data in deciles provided similar results (Supplementary Table S2 and Figure S3, available at https://doi.org/10.1016/j.esmoop.2024.103963).

**Table 2:** Invasive disease-free survival rates by IHC3 score quartiles (IHC3-Q1 to IHC3-Q4)

Patients	IHC3-score	At risk, *n*	Events, *n*	Survival rates		
				2-year	5-year	10-year
All patients	IHC3-Q1 (<81)	594	38	0.98 (0.97-0.99)	0.95 (0.93-0.97)	0.88 (0.85-0.92)
	IHC3-Q2 (81-122)	585	46	0.99 (0.98-1.00)	0.94 (0.91-0.96)	0.85 (0.80-0.89)
	IHC3-Q3 (122-172)	592	49	0.99 (0.98-1.00)	0.93 (0.91-0.96)	0.84 (0.80-0.89)
	IHC3-Q4 (≥172)	588	102	0.94 (0.92-0.96)	0.84 (0.80-0.87)	0.70 (0.64-0.76)
Patients with chemotherapy	IHC3-Q1 (<81)	49	4	0.94 (0.87-1.00)	0.94 (0.87-1.00)	0.91 (0.83-1.00)
	IHC3-Q2 (81-122)	94	16	1.00 (1.00-1.00)	0.88 (0.80-0.96)	0.76 (0.66-0.87)
	IHC3-Q3 (122-172)	164	18	0.99 (0.97-1.00)	0.92 (0.87-0.97)	0.83 (0.76-0.91)
	IHC3-Q4 (≥172)	352	59	0.94 (0.91-0.96)	0.84 (0.80-0.88)	0.74 (0.68-0.81)
Patients without chemotherapy	IHC3-Q1 (<81)	545	34	0.98 (0.97-0.99)	0.96 (0.93-0.98)	0.88 (0.84-0.92)
	IHC3-Q2 (81-122)	491	30	0.99 (0.98-1.00)	0.95 (0.92-0.97)	0.87 (0.82-0.92)
	IHC3-Q3 (122-172)	428	31	0.99 (0.98-1.00)	0.94 (0.91-0.97)	0.85 (0.80-0.90)
	IHC3-Q4 (≥172)	236	43	0.96 (0.93-0.98)	0.84 (0.78-0.90)	0.62 (0.53-0.73)

Survival rates are presented as a percentage in its decimal form.

IHC, immunohistochemical.

A sensitivity analysis with DDFS as outcome showed similar results to iDFS (Supplementary Table S3 and Figure S4, available at https://doi.org/10.1016/j.esmoop.2024.103963). Five-year DDFS rate was lowest in IHC3-Q4 [0.86 (95% CI 0.83-0.90)] versus IHC3-Q1: 0.97 (95% CI 0.95-0.99), IHC3-Q2: 0.95 (95% CI 0.93-0.97), and IHC3-Q3: 0.96 (95% CI 0.94-0.98). In addition, subgroups based on chemotherapy treatment also behaved similarly for DDFS as for iDFS (Supplementary Table S3 and Figure S4, available at https://doi.org/10.1016/j.esmoop.2024.103963).

### Association between IHC3 score and OS

**Figure 2:**
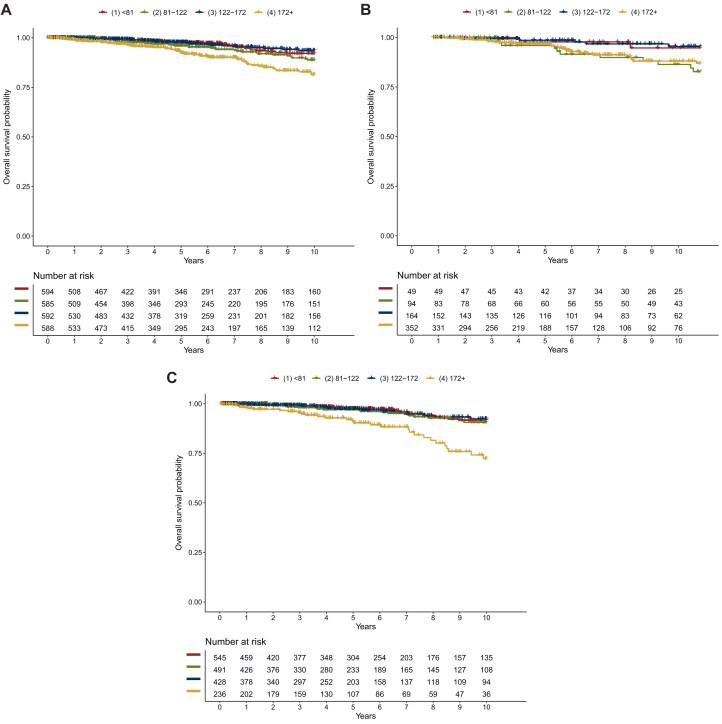
**IHC3 and overall survival.** Overall survival curves by IHC3 score quartiles (A) in the total patient population, (B) in patients treated with chemotherapy and (C) in patients who did not receive chemotherapy. Red line: IHC3-Q1, green line: IHC3-Q2, blue line: IHC3-Q3: yellow line: IHC3-Q4.

Equivalent to iDFS, patients in IHC3-Q4 had the worst prognosis regarding OS [5-year OS: IHC-Q4 0.92 (95% CI 0.90-0.95); IHC-Q3 0.98 (95% CI 0.96-0.99); IHC-Q2 0.96 (95% CI 0.94-0.98); IHC-Q1 0.97 (95% CI 0.96-0.99)] (Table 3 and Figure 2). OS in patients who were not treated with chemotherapy was similar to that in the general population, with IHC3-Q4 having the worst prognosis and IHC3-Q1, -Q2 and -Q3 having better survival rates (Figure 2). In patients treated with chemotherapy, the worst prognosis was observed in IHC3-Q4 and IHC3-Q2 (Figure 2B). Results with IHC3 decile groups were similar (Supplementary Table S4 and Figure S5, available at https://doi.org/10.1016/j.esmoop.2024.103963).

**Table 3:** Overall survival rates by IHC3 score quartiles (IHC3-Q1 to IHC3-Q4)

Patients	IHC3-score	At risk, *n*	Events, *n*	Survival rates		
				2-year	5-year	10-year
All patients	IHC3-Q1 (<81)	594	24	0.99 (0.98-1.00)	0.97 (0.96-0.99)	0.92 (0.89-0.95)
	IHC3-Q2 (81-122)	585	32	1.00 (0.99-1.00)	0.96 (0.94-0.98)	0.89 (0.85-0.93)
	IHC3-Q3 (122-172)	592	20	0.99 (0.98-1.00)	0.98 (0.96-0.99)	0.94 (0.91-0.96)
	IHC3-Q4 (≥172)	588	55	0.98 (0.97-0.99)	0.92 (0.90-0.95)	0.81 (0.77-0.86)
Patients with chemotherapy	IHC3-Q1 (<81)	49	2	1.00 (1.00-1.00)	0.98 (0.94-1.00)	0.95 (0.88-1.00)
	IHC3-Q2 (81-122)	94	11	1.00 (1.00-1.00)	0.92 (0.85-0.98)	0.83 (0.74-0.93)
	IHC3-Q3 (122-172)	164	5	0.99 (0.98-1.00)	0.99 (0.97-1.00)	0.95 (0.92-1.00)
	IHC3-Q4 (≥172)	352	26	0.98 (0.97-1.00)	0.93 (0.90-0.97)	0.87 (0.82-0.92)
Patients without chemotherapy	IHC3-Q1 (<81)	545	22	0.99 (0.98-1.00)	0.97 (0.96-0.99)	0.92 (0.88-0.95)
	IHC3-Q2 (81-122)	491	21	0.99 (0.99-1.00)	0.97 (0.95-0.99)	0.91 (0.87-0.95)
	IHC3-Q3 (122-172)	428	15	0.99 (0.98-1.00)	0.97 (0.95-0.99)	0.92 (0.88-0.96)
	IHC3-Q4 (≥172)	236	29	0.97 (0.95-0.99)	0.91 (0.87-0.96)	0.72 (0.63-0.83)

IHC, immunohistochemical.

## Discussion

In this retrospective analysis, we evaluated the prognostic impact of the IHC3 score along with clinical factors (as developed by Cuzick et al.^18^) in patients with node-negative HRpos/HER2neg early-stage breast cancer. We could show that patients can be allocated to prognostic categories based on the IHC3 score, with the highest IHC3 score being associated with the poorest prognosis.

Several studies evaluated the prognostic value of IHC3/4 and compared it to other prognostic models including multigene assays.^18^, ^19^, ^20^ In the transATAC subset of the ATAC study,^24^ the potential improvement in prognosis prediction of the IHC4 model and the 21-gene assay (recurrence score, RS21) in combination with clinical parameters over conventional clinical parameters alone was assessed. In this cohort, prognosis prediction with IHC4 and RS21 was comparable.^18^ In a larger comparative study within transATAC, six different prognostic models were compared such as clinical treatment score (CTS),^18^ IHC4,^18^ RS21,^18^ breast cancer index (BCI),^25^ Prosigna risk of recurrence (ROR)^19^ and Endopredict (EPclin).^26^ All prognostic models provided significant prognostic information during the complete 10-year follow-up. However, BCI, ROR and EPclin performed best, specifically for the prediction of recurrences between years 5 and 10.^20^ In this analysis, IHC4 and CTS scores appeared to outperform RS21 when considering the entire 10-year follow-up period,^20^ which indicates that this simple and easily implementable model provides valuable information.

Although our study did not compare the prognostic predictive value of IHC3 plus clinical factors with other models, patients with a more unfavorable prognosis could distinctly be identified. Node-negative patients in the IHC3-Q4 subgroup had a 2-year iDFS rate of 94% and patients in the 10th decile had a 2-year iDFS of 91%, which is comparable to the control arm of the Ki-67-low cohort of the monarchE population (node-positive HRpos/HER2neg early breast cancer with high risk of recurrence).^13^ This demonstrates that node-negative patients with a high IHC3 score fall within the same prognostic category as the node-positive monarchE subgroups with a more favorable prognosis, which could indicate the potential need for therapy adjustment in this patient population. In NATALEE, which also included node-negative patients with increased risk factors, subgroup analyses found no differences based on Ki-67 status or nodal status.^27^ Of note, using Ki-67 alone to define a high-risk population has been criticized for a lack of reproducibility.^28^ IHC3 could therefore provide a more robust assessment of prognosis, integrating not only Ki-67, but also ER, PR and clinical factors. In our population, IHC3 quartiles could allocate breast cancer patients in prognostic categories for iDFS, DDFS and OS.

Although no formal statistical comparisons between patients receiving chemotherapy versus not receiving chemotherapy were made, the prognostic prediction of IHC3 plus clinical factors in patients treated with chemotherapy is interesting. While patients with the highest IHC3 score (IHC3-Q4) had the worst prognosis and those with the lowest IHC3 score (IHC3-Q1) the best prognosis, IHC3-Q2 and IHC3-Q3 appeared inverted: patients in IHC3-Q2 had a poorer prognosis than patients in IHC3-Q3. Potentially, this could be the consequence of different responsiveness to the given chemotherapy. Indeed, higher Ki-67 values are known to be associated with a higher chemotherapy efficacy in the neoadjuvant setting.^29^ In addition, tumors with low HR expression respond better to chemotherapy than those with a higher expression.^30^ In our study, IHC3 positively correlated with Ki-67 expression and inversely correlated with HR expression. Therefore, chemotherapy might be more effective in patients with higher IHC3 score than in those with lower IHC3 scores. A neoadjuvant study could indeed show an increase in chemotherapy response with increasing IHC4 score.^31^ Therefore, the prognostic behavior of patients receiving chemotherapy could reflect both the prognostic value of the IHC3 score and the response to the given chemotherapy.

Our study has some limitations. Although the quality of the assessed biomarkers has been demonstrated to be useful for the prediction of therapy efficacy and prognostication,^29^^,^^32^ reproducibility of our results might be a concern due to the monocentric nature of this study. Furthermore, despite having a large overall patient population, the number of patients in the chemotherapy and low IHC3 score subgroups was rather low, limiting strong interpretation of these results.

In conclusion, IHC3 is able to define prognostic groups in patients with node-negative, HRpos/HER2neg early breast cancer. Node-negative patients with a high IHC3 score had the worst prognosis, which was comparable with that of node-positive patients described in recent trials. As such, further investigation on the potential use of innovative therapies, e.g. CDK4/6 inhibitors and SERDs, for patients with node-negative disease with an unfavorable prognosis is warranted. IHC3 could be a simple and cost-effective tool to determine this prognosis.
