# Supplementary material for: Prognosis prediction with the IHC3 score in patients with node-negative, hormone receptor-positive, HER2-negative early breast cancer
Source: ESMO Open. 2024 Oct 26;9(11):103963. doi: 10.1016/j.esmoop.2024.103963 (PMC11558624; doi:10.1016/j.esmoop.2024.103963)
Supplement: Supplementary Material [file mmc1.docx]

**Supplementary material for:**

**Prognosis prediction with the IHC3 score in patients with node-negative, hormone receptor-positive, HER2-negative early breast cancer**

Katharina Seitz ^1,2*^, Chloë Goossens ^1,2*^, Hanna Huebner ^1,2^, Paul Gass ^1,2^, Sabrina Uhrig ^1,2^, Felix Heindl ^1,2^, Julius Emons ^1,2^, Matthias Ruebner ^1,2^, Daniel Anetsberger ^1,2^, Arndt Hartmann ^2,3^, Matthias W. Beckmann ^1,2^, Ramona Erber ^2,3^, Carolin C. Hack ^1,2^, Peter A. Fasching ^1,2^, Lothar Häberle^1,2,4^

^1^ Department of Gynecology and Obstetrics, Universitätsklinikum Erlangen, Friedrich-Alexander-Universität Erlangen-Nürnberg (FAU), Erlangen, Germany

^2^ Comprehensive Cancer Center Erlangen-EMN (CCC-ER-EMN), Erlangen, Germany

^3^ Institute of Pathology, Universitätsklinikum Erlangen, Friedrich-Alexander-Universität Erlangen-Nürnberg (FAU), Erlangen, Germany

^4^ Biostatistics Unit, Department of Gynecology and Obstetrics, Universitätsklinikum Erlangen, Friedrich-Alexander-Universität Erlangen-Nürnberg (FAU), Erlangen, Germany

*these authors contributed equally

**Supplementary Table 1.** Characteristics of study population by IHC3 score deciles (IHC3-D1 – IHC3-D10). Data is presented as either mean (standard deviation) or N (%)

| **Characteristic** |  | **IHC3-score** | | | | | | | | | |
| --- | --- | --- | --- | --- | --- | --- | --- | --- | --- | --- | --- |
|  |  | **IHC3-D1 (<40) (N = 237)** | **IHC3-D2 (40-72) (N = 237)** | **IHC3-D3 (72-90) (N = 240)** | **IHC3-D4 (90-105) (N = 221)** | **IHC3-D5 (105-122) (N = 244)** | **IHC3-D6 (122-142) (N = 242)** | **IHC3-D7 (142-160) (N = 224)** | **IHC3-D8 (160-186) (N = 243)** | **IHC3-D9 (186-224)  (N = 235)** | **IHC3-D10 (≥224) (N = 236)** |
|  |  |  |  |  |  |  |  |  |  |  |  |
| Age at diagnosis (years) |  | 58.2 (9.5) | 58.1 (10.7) | 58.9 (10.8) | 59.7 (11.0) | 59.3 (12.3) | 59.0 (11.9) | 62.1 (11.3) | 61.2 (12.0) | 59.6 (12.2) | 57.0 (14.4) |
| ER expression (0-100%) |  | 88.0 (9.8) | 85.9 (13.3) | 85.3 (15.0) | 86.0 (13.0) | 83.9 (13.8) | 81.1 (19.4) | 82.3 (18.4) | 80.8 (19.6) | 78.9 (22.7) | 52.6 (37.0) |
| PR expression (0-100%) |  | 81.5 (15.5) | 71.1 (24.4) | 63.8 (32.4) | 68.9 (27.3) | 62.3 (29.4) | 56.5 (31.4) | 48.3 (33.3) | 32.8 (31.4) | 31.4 (32.3) | 15.4 (23.3) |
| Ki-67 expression (0-100%) |  | 6.0 (3.9) | 8.6 (5.7) | 9.4 (6.4) | 12.4 (7.3) | 13.6 (9.1) | 15.1 (10.6) | 19.0 (13.0) | 21.0 (13.7) | 27.1 (15.8) | 42.5 (24.3) |
| Tumor stage | T1 | 236 (99.6) | 214 (90.3) | 214 (89.2) | 190 (86.0) | 197 (80.7) | 155 (64.0) | 132 (58.9) | 145 (59.7) | 98 (41.7) | 60 (25.4) |
|  | T2 | 1 (0.4) | 23 (9.7) | 26 (10.8) | 31 (14.0) | 47 (19.3) | 86 (35.5) | 91 (40.6) | 91 (37.4) | 118 (50.2) | 110 (46.6) |
|  | T3 | 0 (0.0) | 0 (0.0) | 0 (0.0) | 0 (0.0) | 0 (0.0) | 0 (0.0) | 1 (0.4) | 6 (2.5) | 15 (6.4) | 42 (17.8) |
|  | T4 | 0 (0.0) | 0 (0.0) | 0 (0.0) | 0 (0.0) | 0 (0.0) | 1 (0.4) | 0 (0.0) | 1 (0.4) | 4 (1.7) | 24 (10.2) |
| Grading | G1 | 232 (97.9) | 166 (70.0) | 99 (41.2) | 45 (20.4) | 35 (14.3) | 19 (7.9) | 9 (4.0) | 5 (2.1) | 2 (0.9) | 1 (0.4) |
|  | G2 | 4 (1.7) | 71 (30.0) | 141 (58.8) | 175 (79.2) | 202 (82.8) | 210 (86.8) | 188 (83.9) | 186 (76.5) | 117 (49.8) | 80 (33.9) |
|  | G3 | 1 (0.4) | 0 (0.0) | 0 (0.0) | 1 (0.5) | 7 (2.9) | 13 (5.4) | 27 (12.1) | 52 (21.4) | 116 (49.4) | 155 (65.7) |
| Chemotherapy | No | 219 (92.4) | 215 (90.7) | 217 (90.4) | 191 (86.4) | 194 (79.5) | 179 (74.0) | 160 (71.4) | 159 (65.4) | 103 (43.8) | 63 (26.7) |
|  | Yes | 18 (7.6) | 22 (9.3) | 23 (9.6) | 30 (13.6) | 50 (20.5) | 63 (26.0) | 64 (28.6) | 84 (34.6) | 132 (56.2) | 173 (73.3) |
|  |  |  |  |  |  |  |  |  |  |  |  |

*ER* estrogen receptor, *PR* progesterone receptor

**Supplementary Table 2**: Invasive disease-free survival rates by IHC3 score deciles (IHC3-D1 – IHC3-D10).

| **IHC3-score** | **At risk** | **Events** | **Survival rates** | | |
| --- | --- | --- | --- | --- | --- |
|  |  |  | **2-year** | **5-year** | **10-year** |
| IHC3-D1 (<40) | 237 | 15 | 0.98 (0.97, 1.00) | 0.95 (0.91, 0.98) | 0.89 (0.84, 0.95) |
| IHC3-D2 (40–72) | 237 | 15 | 0.98 (0.96, 1.00) | 0.98 (0.95, 1.00) | 0.87 (0.80, 0.94) |
| IHC3-D3 (72–90) | 240 | 16 | 0.98 (0.96, 1.00) | 0.94 (0.91, 0.98) | 0.87 (0.81, 0.94) |
| IHC3-D4 (90-105) | 221 | 15 | 0.98 (0.97, 1.00) | 0.94 (0.91, 0.98) | 0.88 (0.81, 0.94) |
| IHC3-D5 (105–122) | 244 | 23 | 0.99 (0.99, 1.00) | 0.91 (0.87, 0.96) | 0.81 (0.74, 0.89) |
| IHC3-D6 (122–142) | 242 | 17 | 0.98 (0.96, 1.00) | 0.95 (0.92, 0.98) | 0.88 (0.82, 0.94) |
| IHC3-D7 (142–160) | 224 | 17 | 1.00 (1.00, 1.00) | 0.93 (0.89, 0.97) | 0.86 (0.79, 0.93) |
| IHC3-D8 (160–186) | 243 | 30 | 0.98 (0.96, 1.00) | 0.90 (0.86, 0.95) | 0.75 (0.67, 0.84) |
| IHC3-D9 (186–224) | 235 | 41 | 0.96 (0.93, 0.99) | 0.84 (0.79, 0.89) | 0.72 (0.64, 0.80) |
| IHC3-D10 (≥224) | 236 | 46 | 0.91 (0.88, 0.95) | 0.81 (0.75, 0.87) | 0.66 (0.57, 0.76) |
|  |  |  |  |  |  |

**Supplementary Table 3.** Distant disease-free survival rates by IHC3 score quartiles (IHC3-Q1 – IHC3-Q4).

| **Patients** | **IHC3-score** | **At risk** | **Events** | **Survival rates** | | |
| --- | --- | --- | --- | --- | --- | --- |
|  |  |  |  | **2-year** | **5-year** | **10-year** |
| All patients | IHC3-Q1 (<81) | 594 | 29 | 0.99 (0.98, 1.00) | 0.97 (0.95, 0.99) | 0.90 (0.87, 0.94) |
|  | IHC3-Q2 (81–122) | 585 | 37 | 1.00 (0.99, 1.00) | 0.95 (0.93, 0.97) | 0.87 (0.83, 0.91) |
|  | IHC3-Q3 (122–172) | 592 | 32 | 0.99 (0.98, 1.00) | 0.96 (0.94, 0.98) | 0.90 (0.86, 0.93) |
|  | IHC3-Q4 (≥172) | 588 | 84 | 0.96 (0.94, 0.98) | 0.86 (0.83, 0.90) | 0.74 (0.69, 0.80) |
| Patients with chemotherapy | IHC3-Q1 (<81) | 49 | 3 | 0.96 (0.90, 1.00) | 0.96 (0.90, 1.00) | 0.93 (0.86, 1.00) |
|  | IHC3-Q2 (81–122) | 94 | 13 | 1.00 (1.00, 1.00) | 0.89 (0.82, 0.97) | 0.80 (0.71, 0.91) |
|  | IHC3-Q3 (122–172) | 164 | 14 | 0.99 (0.97, 1.00) | 0.93 (0.89, 0.98) | 0.87 (0.81, 0.94) |
|  | IHC3-Q4 (≥172) | 352 | 46 | 0.95 (0.93, 0.98) | 0.87 (0.83, 0.91) | 0.79 (0.73, 0.85) |
| Patients without chemotherapy | IHC3-Q1 (<81) | 545 | 26 | 0.99 (0.98, 1.00) | 0.97 (0.95, 0.99) | 0.90 (0.86, 0.94) |
|  | IHC3-Q2 (81–122) | 491 | 24 | 0.99 (0.99, 1.00) | 0.96 (0.94, 0.98) | 0.89 (0.85, 0.94) |
|  | IHC3-Q3 (122–172) | 428 | 18 | 0.99 (0.98, 1.00) | 0.97 (0.95, 0.99) | 0.91 (0.87, 0.95) |
|  | IHC3-Q4 (≥172) | 236 | 38 | 0.97 (0.94, 0.99) | 0.85 (0.80, 0.91) | 0.66 (0.56, 0.77) |

**Supplementary Table 4**: Overall survival rates by IHC3 score deciles (IHC3-D1 – IHC3-D10).

| **IHC3-score** | **At risk** | **Events** | **Survival rates** | | |
| --- | --- | --- | --- | --- | --- |
|  |  |  | **2-year** | **5-year** | **10-year** |
| IHC3-D1 (<40) | 237 | 9 | 1.00 (1.00, 1.00) | 0.97 (0.95, 1.00) | 0.93 (0.88, 0.97) |
| IHC3-D2 (40–72) | 237 | 11 | 0.99 (0.98, 1.00) | 0.98 (0.96, 1.00) | 0.90 (0.85, 0.96) |
| IHC3-D3 (72–90) | 240 | 10 | 0.99 (0.98, 1.00) | 0.98 (0.97, 1.00) | 0.90 (0.84, 0.97) |
| IHC3-D4 (90-105) | 221 | 11 | 0.99 (0.98, 1.00) | 0.96 (0.93, 0.99) | 0.90 (0.85, 0.96) |
| IHC3-D5 (105–122) | 244 | 15 | 0.99 (0.99, 1.00) | 0.94 (0.90, 0.98) | 0.88 (0.82, 0.94) |
| IHC3-D6 (122–142) | 242 | 7 | 0.98 (0.96, 1.00) | 0.98 (0.96, 1.00) | 0.96 (0.92, 0.99) |
| IHC3-D7 (142–160) | 224 | 8 | 1.00 (1.00, 1.00) | 0.98 (0.96, 1.00) | 0.92 (0.87, 0.98) |
| IHC3-D8 (160–186) | 243 | 13 | 0.99 (0.98, 1.00) | 0.96 (0.93, 0.99) | 0.90 (0.84, 0.96) |
| IHC3-D9 (186–224) | 235 | 19 | 0.99 (0.98, 1.00) | 0.95 (0.91, 0.98) | 0.85 (0.78, 0.92) |
| IHC3-D10 (≥224) | 236 | 28 | 0.97 (0.94, 0.99) | 0.89 (0.84, 0.94) | 0.75 (0.66, 0.85) |
|  |  |  |  |  |  |

**
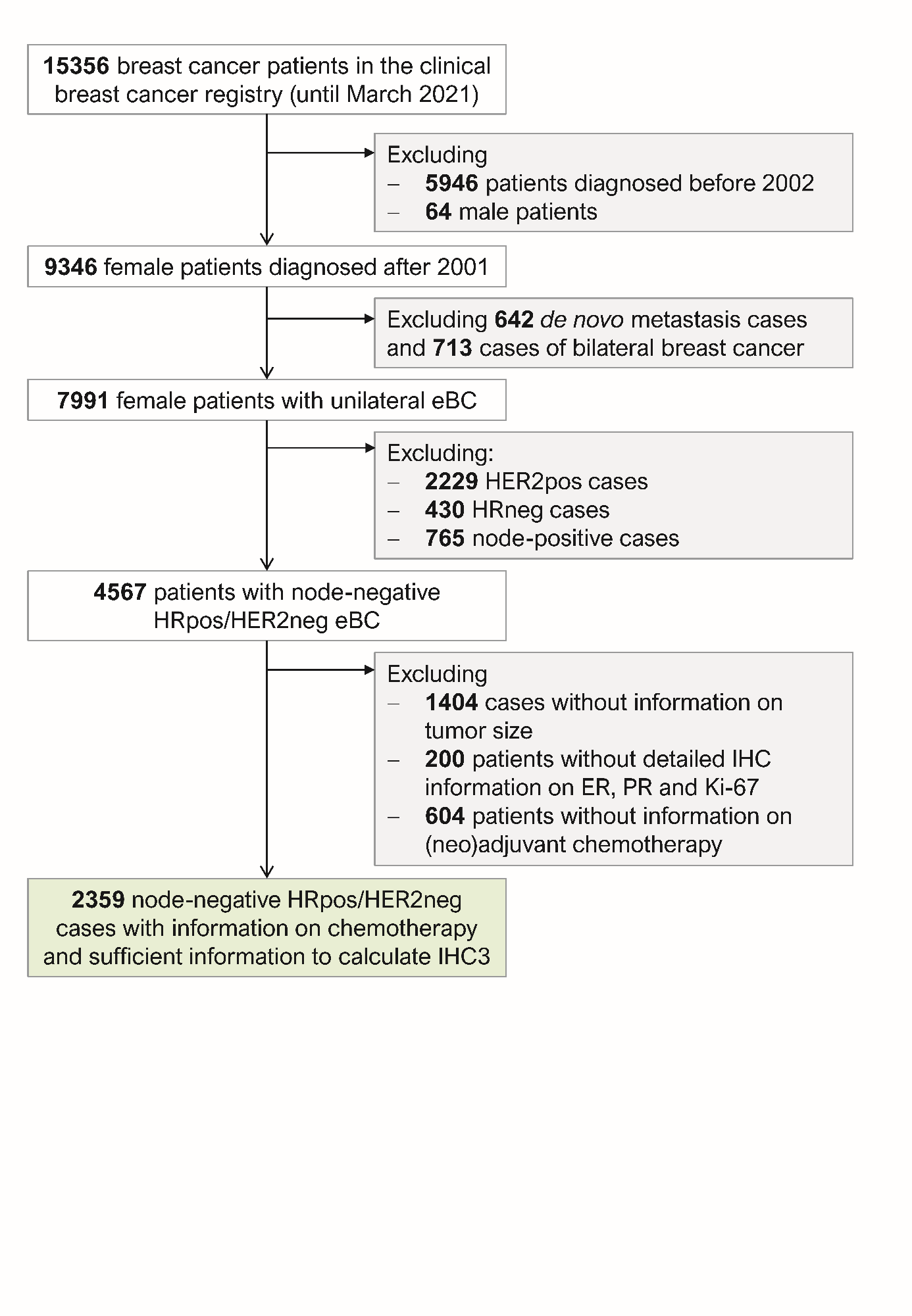
**

**Supplementary Figure 1**: Patient selection. HR: hormone receptor; pos: positive; neg: negative; ER: estrogen receptor; PR: progesterone receptor; IHC: immunohistochemistry; eBC: early breast cancer


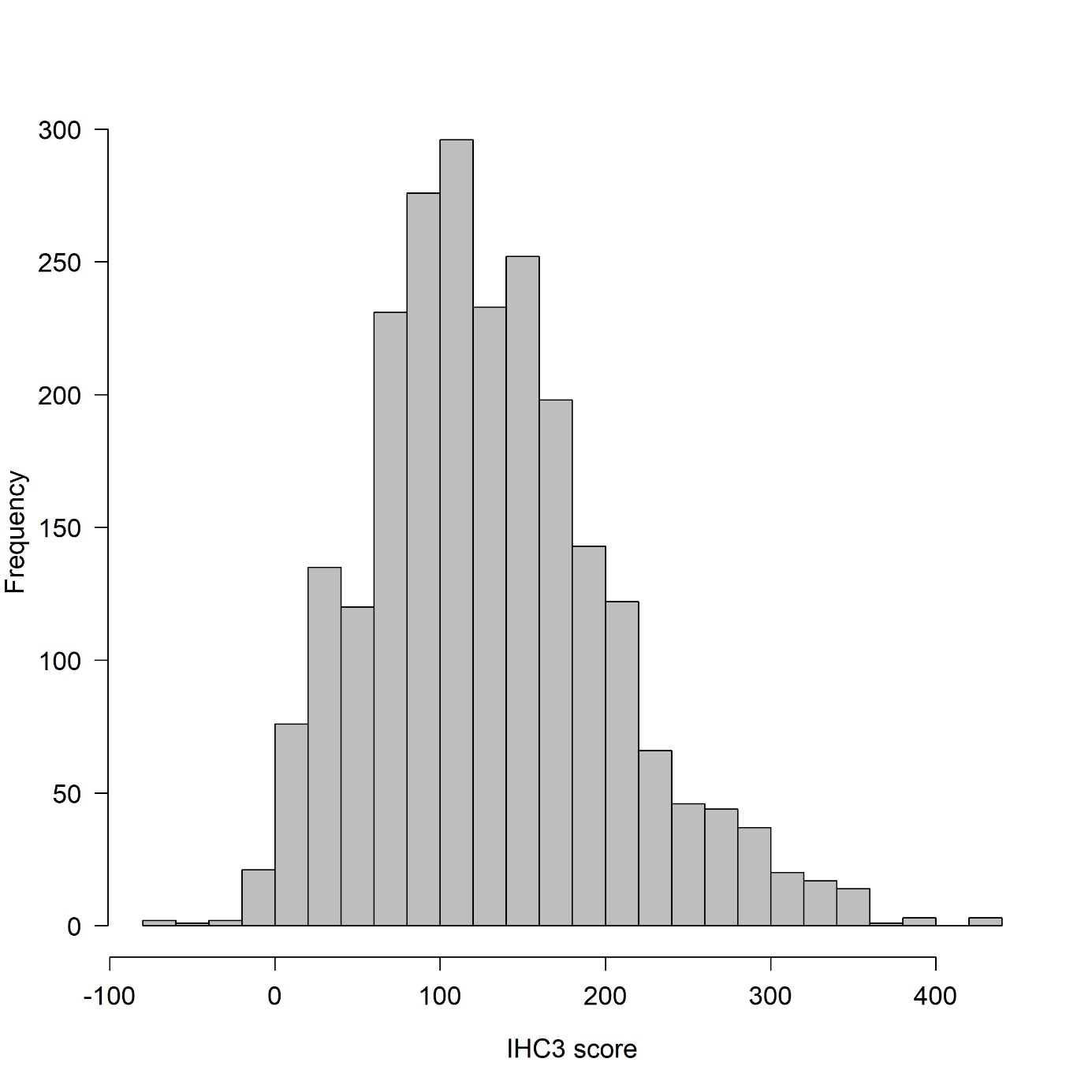


**Supplementary Figure 2.** Distribution of the IHC3 score in the study population.


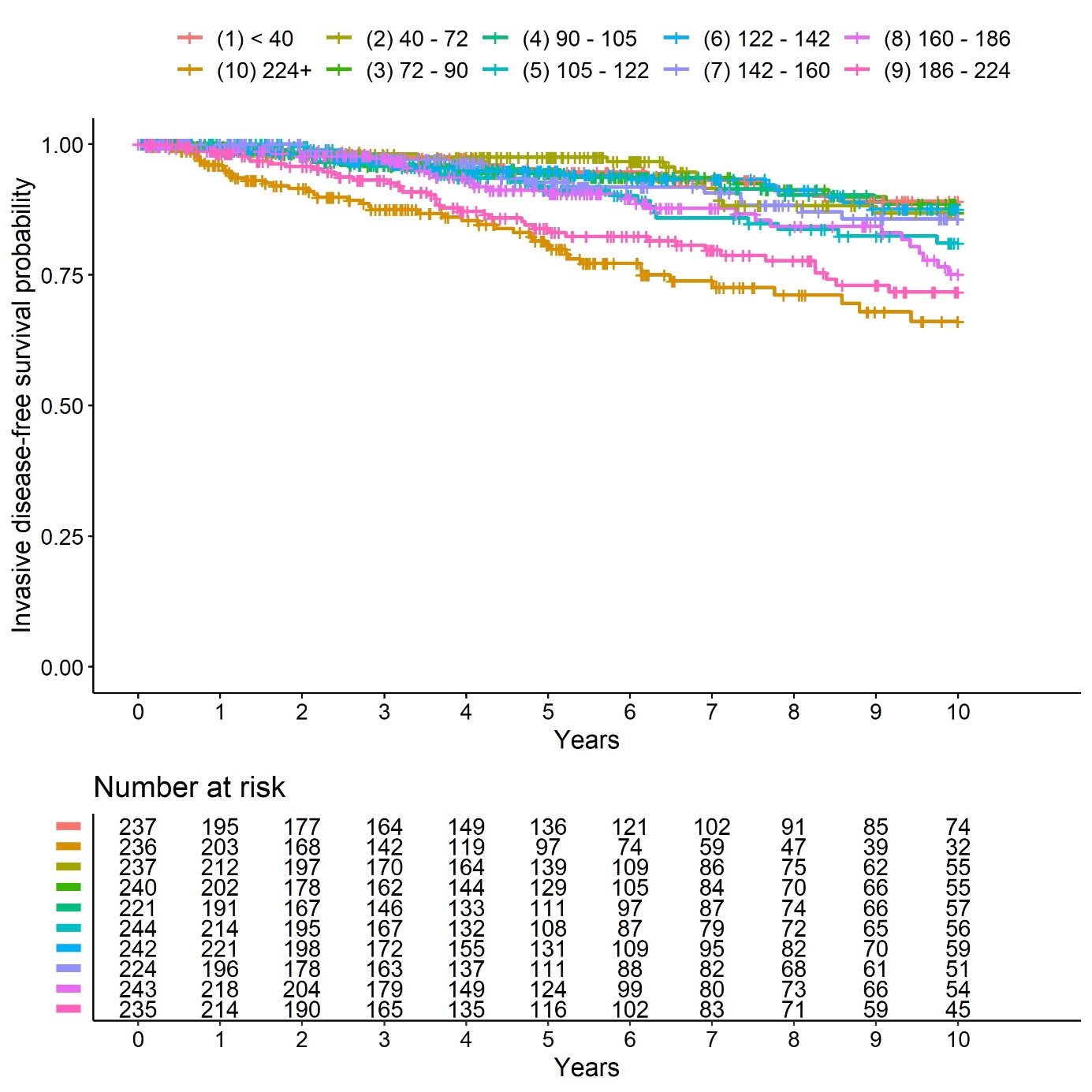


**Supplementary Figure 3**: Invasive disease-free survival curves by IHC3-score deciles.

| **A** total patient population | |
| --- | --- |
| **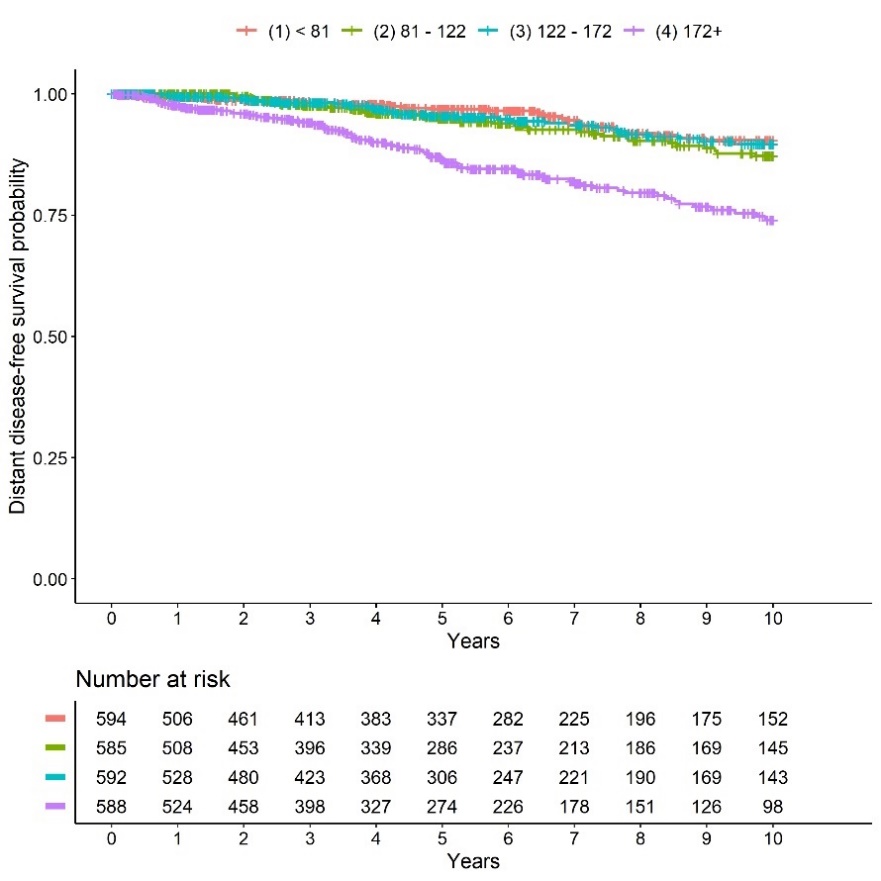** | |
| **B** patients treated with chemotherapy | **C** patients not treated with chemotherapy |
| **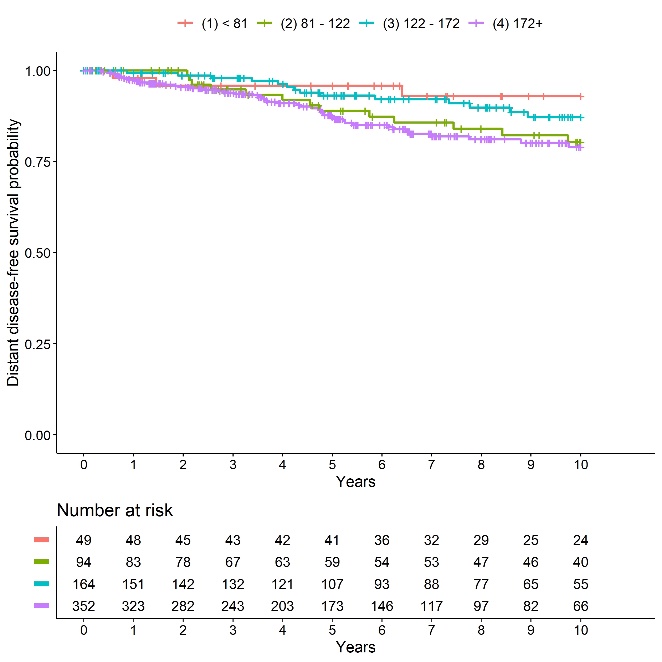** | **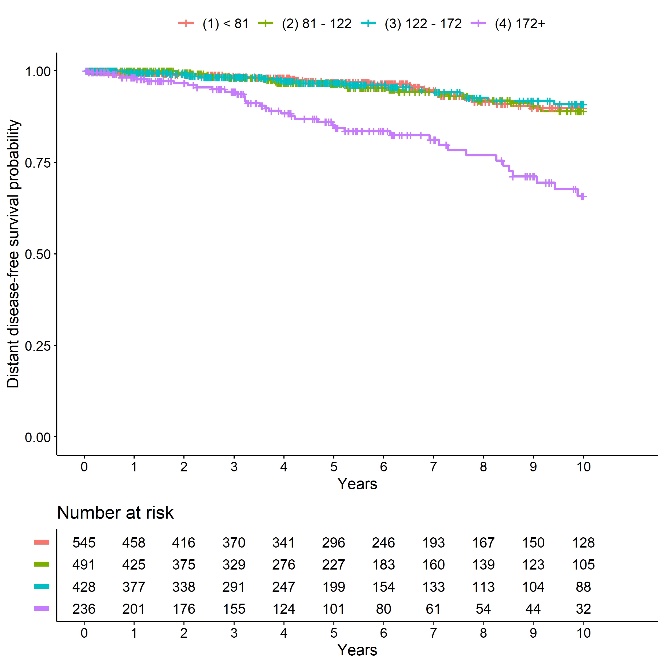** |

**Supplementary Figure 4**: Distant disease-free survival curves by IHC3-score quartiles, in a) the total patient population, b) in patients treated with chemotherapy and c) in patients not treated with chemotherapy. Red graph: IHC3-Q1, green graph: IHC3-Q2, blue graph: IHC3-Q3: purple graph: IHC3-Q4


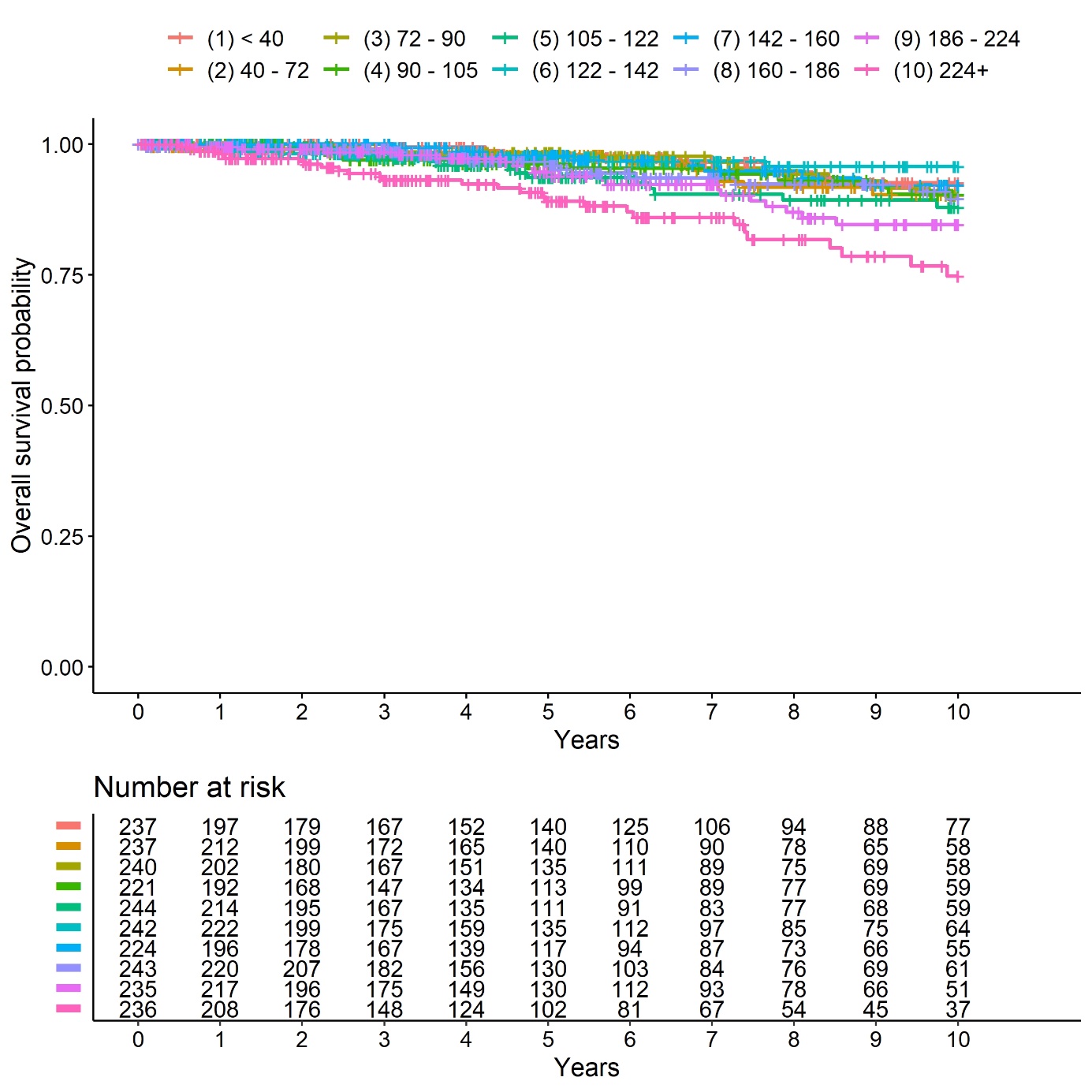


**Supplementary Figure 5**: Overall survival curves by IHC3-score deciles.
